# The Population Structure of *Glossina palpalis gambiensis* from Island and Continental Locations in Coastal Guinea

**DOI:** 10.1371/journal.pntd.0000392

**Published:** 2009-03-17

**Authors:** Philippe Solano, Sophie Ravel, Jeremy Bouyer, Mamadou Camara, Moise S. Kagbadouno, Naomi Dyer, Laetitia Gardes, Damien Herault, Martin J. Donnelly, Thierry De Meeûs

**Affiliations:** 1 CIRDES/IRD UMR 177 IRD-CIRAD, Bobo-Dioulasso, Burkina Faso; 2 IRD, UMR 177 IRD-CIRAD, LRCT Campus International de Baillarguet, Montpellier, France; 3 CIRDES/CIRAD UMR 15 CIRAD-INRA, Bobo-Dioulasso, Burkina Faso; 4 PNLTHA, Ministère de la Santé, Conakry, Guinea; 5 Vector Group, Liverpool School of Tropical Medicine, Liverpool, United Kingdom; 6 GEMI, UMR 2724 IRD/CNRS, Montpellier, France; Liverpool School of Tropical Medicine, United Kingdom

## Abstract

**Background:**

We undertook a population genetics analysis of the tsetse fly *Glossina palpalis gambiensis*, *a* major vector of sleeping sickness in West Africa, using microsatellite and mitochondrial DNA markers. Our aims were to estimate effective population size and the degree of isolation between coastal sites on the mainland of Guinea and Loos Islands. The sampling locations encompassed Dubréka, the area with the highest Human African Trypanosomosis (HAT) prevalence in West Africa, mangrove and savannah sites on the mainland, and two islands, Fotoba and Kassa, within the Loos archipelago. These data are discussed with respect to the feasibility and sustainability of control strategies in those sites currently experiencing, or at risk of, sleeping sickness.

**Principal Findings:**

We found very low migration rates between sites except between those sampled around the Dubréka area that seems to contain a widely dispersed and panmictic population. In the Kassa island samples, various effective population size estimates all converged on surprisingly small values (10<*N_e_*<30) that suggest either a recent bottleneck, and/or other biological or ecological factors such as strong variance in the reproductive success of individuals.

**Conclusion/Significance:**

Whatever their origin, the small effective population sizes suggest high levels of inbreeding in tsetse flies within the island samples in marked contrast to the large diffuse deme in Dubréka zones. We discuss how these genetic results suggest that different tsetse control strategies should be applied on the mainland and islands.

## Author Summary

Guinea is the country with the highest prevalence of sleeping sickness in West Africa, and we undertook a population genetics analysis there of the most dangerous tsetse fly species of West Africa, *Glossina palpalis gambiensis*. Our aims were to estimate effective population size and the degree of isolation between coastal sites on the mainland of Guinea (including Dubréka, a highly prevalent sleeping sickness focus) and Loos Islands in order to get the most possible accurate vision of feasibility and sustainability of anti-tsetse strategies of these sites. We found very low migration rates of tsetse between sites except between those situated in the Dubréka area, which seems to contain a widely distributed panmictic tsetse population (i.e. a population where mating occurs at random). Effective population sizes on Loos islands estimated with various techniques all converged to surprisingly small values. These values might be explained by a recent decrease in tsetse numbers on Kassa Island due to bauxite mining activities. But on the other sites, other explanations have to be found, including possible variance in reproductive success. Our genetic results suggest that different control strategies should be advised on the mainland (reduction in tsetse densities, no elimination) compared to the islands (total elimination feasible). This approach could be extended to many areas where vector control of Human and Animal Trypanosomoses is contemplated.

## Introduction

Mating pattern, population size and migration represent key factors determining the population genetic structure of organisms and shape the evolutionary of species [1]–[4]. Estimating these parameters is a major objective of population and conservation genetics [5]–[7]. Molecular markers are useful for estimating these parameters without the need for costly capture-mark-release-recapture (MRR) studies; particularly for organisms such as parasites and their vectors, where techniques such as MRR are difficult, impossible or unethical to apply [2],[4]. Furthermore a detailed understanding of parasite and vector population dynamics is crucial for effective sustainable control [2], [8]–[10].

The World Health Organisation recently launched a Human African Trypanosomosis (HAT, or sleeping sickness) elimination programme to counter the recent decline in case detection and treatment, notably in Central Africa [11]–[12]. However the situation in West Africa and the epidemiology of HAT is less well described. Guinea (especially the coastal area) is believed to be the country most affected by this disease [13]. Guinea has a long history of sleeping sickness, which was particularly prevalent in the years 1930–40 [14]. Current data show prevalences of between 2 and 5% in villages in the coastal mangrove area (Dubreka focus) [15]. This coastal area is composed of mangrove on the coastal margins and savannah inland. Offshore but in close proximity to the Dubreka focus lie the Loos Islands. These islands, physically separated from the mainland about 5000 years ago (D. Bazzo, pers. com.) are known to harbour tsetse flies (*Glossina palpalis gambiensis* (Diptera: Glossinidae), the main vector of HAT in West Africa and the Guinean National Control Programme against HAT recently launched an tsetse elimination programme on the archipelago. To facilitate the work of the elimination programme we used microsatellite and mitochondrial markers to address the following questions which are key to the successful control of tsetse: What is the effective population size in this tsetse species? What is the extent of genetic differentiation between mainland sites, between the islands and the mainland, and between the different islands of Loos archipelago? By answering these questions we hope to improve the design of control strategies in the region, especially with respect to designing and implementing area wide strategies which must target genetically isolated populations if elimination is the objective [16]. Our results suggest that tsetse elimination is a feasible strategy on the Loos islands given both the genetic isolation between island and mainland populations and the small total surface to be controlled, but transmission reduction rather than elimination is more advisable for mainland tsetse populations.

## Methods

### Study area

**Figure 1:**
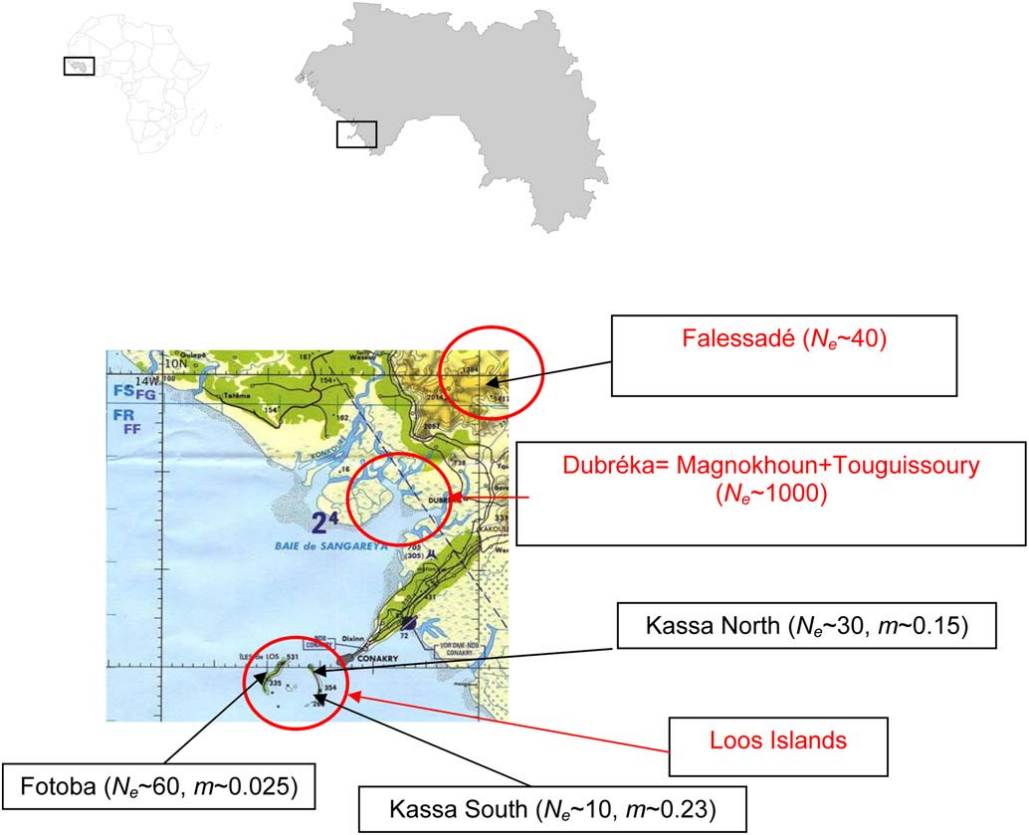
Geographic location of the studied samples. Samples from Loos islands include Fotoba (the western island) and Kassa. On the mainland, samples come from the mangrove (Magnokhoun and Touguissoury) and the savannah (Falessadé). *N_e_* is the order of magnitude for effective population sizes as in Table 6 and *m* is migration rate averaged from Table 5 for Loos Island sites.

The Loos islands are a small archipelago of five islands separated from the mainland of Guinea and the capital Conakry, by 4 km of sea. Three of the islands are inhabited, Kassa, Fotoba and Room in order of decreasing population size, with a total of around 7,000 inhabitants. On Kassa Island, two areas were sampled for tsetse, one in the north and one in the south. The biggest focus of HAT in Guinea, Dubréka, is on the mainland in a mangrove some 30 km distant from the Loos Islands. The area around Dubreka is characterised by coastal mangrove, with anthropic Guinean savannah, and permanently or temporarily inundated areas. Near the town of Dubréka (25,000 inhabitants), people live in villages of between 300 to 2,000 inhabitants. The main economic activities include fishing, salt extraction, and agriculture (palm and mango plantations, rice and food crops). In the Dubréka area, tsetse were sampled in 12 sites from two main areas: Touguissoury (five sites), in the mangrove habitat and accessible only by boat, and Magnokhoun (six sites) which is at the boundary between mangrove and savannah. A savannah area comprising a forest gallery bordering a water course was also sampled: Falessadé (one site), 30 km from the mangrove areas of Magnokhoun (see Figure 1).

### Entomological surveys

Tsetse collections were made at each location using Vavoua traps [17]. Collecting cages were changed daily over a period of two to four days, and tsetse were counted and separated by sex. Three legs were removed from each fly and put in individual, labelled, dry eppendorf tubes.

All the continental samples and those from Fotoba Island were taken in 2005. Temporal samples were taken in Loos islands: 2005 and 2006 for Fotoba, 2006 and 2007 for Kassa.

### Microsatellite markers

A total of 195 individuals were used for the genetic analyses at microsatellite loci: 7 males (M) and 15 females (F) in Kassa 2006, 14 M and 11 F in Fotoba 2005, 7F and 14M in Fotoba 2006, 18F and 12M in Kassa 2007, 17M and 15F in Magnokhoun 2005, 17M and 17F in Touguissoury 2005, and 11M and 20F in Falessadé 2005. Ten microsatellite loci were analysed: Gpg55,3 [18], pGp24, pGp 13, pGp11, pGp1 [19], C102, B104, B110 (kindly given by A.S. Robinson), GpCAG [20], and A10 kindly provided by G. Caccone. Locus Gpg55,3 has been reported to be located on the X chromosome [21], and given an absence of heterozygotes on a subsample of males (data not shown), B104, B110, Pgp13 and pgp11 were also interpreted to be located on the X chromosome. For ease of reference we renamed X linked loci XGpg55,3, XB104, XB110, XPgp13 and XpGp11. Because loci A10 and pGp1 were unavailable before 2007, these loci were only used for 2007 sample (Kassa 2007) and thus only influenced local results (Linkage disequilibrium and *F*_IS_ analyses).

In each tube containing three legs of the tsetse, 200 µl of 5% Chelex chelating resin was added [22]–[23]. After incubation at 56°C for one hour, DNA was denatured at 95°C for 30 min. The tubes were then centrifuged at 12,000 g for two min and frozen for later analysis.

The PCR reactions were carried out in a thermocycler (MJ Research, Cambridge, UK) in 10 µl final volume, using 1 µl of the supernatant from the extraction step. After PCR amplification, allele bands were resolved on a 4300 DNA Analysis System (LI-COR,Lincoln, NE) after migration on 96-lane reloadable 6.5% denaturing polyacrylamide gels. This method allows multiplexing of loci by the use of two infrared dyes (IRDye), separated by 100 nm (700 and 800 nm), and read by a two channel detection system that uses two separate lasers and detectors to eliminate errors due to fluorescence overlap. To determine the different allele sizes, a large panel of about 30 size markers was used. These size markers had been previously generated by cloning alleles from individual tsetse flies into pGEM-T Easy Vector (Promega Corporation, Madison, WI, USA). Three clones of each allele were sequenced using the T7 primer and the Big Dye Terminator Cycle Sequencing Ready Reaction Kit (PE Applied Biosystems, Foster City, CA, USA). Sequences were analysed on a PE Applied Biosystems 310 automatic DNA sequencer (PE Applied Biosystems) and the exact size of each cloned allele was determined. PCR products from these cloned alleles were run in the same acrylamide gel as the samples, allowing the allele size of the samples to be determined accurately.

### Data analysis

Linkage disequilibrium between pairs of loci was tested under Fstat 2.9.3.2 [24], updated from [25] by randomising loci (free recombination) with a G (log-likelihood ratio) based test allowing to get, for each pair of loci, a global test across sub-samples. For this analysis, the sub-sample unit was the smallest available one (e.g. the trap in Dubreka). Because this procedure involves multiple testing the *P*-values obtained were adjusted with a sequential Bonferroni procedure [26] (see [2] and references therein for detailed information). A binomial test was used to check if the proportion of significant tests was significantly greater than expected based upon a 5% significance level (see [27]).

Wright's *F*-statistics, the parameters most widely used to describe population structure [28], were initially defined for a three levels hierarchical population structure (individuals, sub-populations and total). In such a structure, three fixation indices or *F*-statistics can be defined: *F*_IS_ is a measure of the inbreeding of individuals (hence I) resulting from the deviation from panmixia (random union of gametes) within each sub-population (hence S). *F*_ST_ is a measure of inbreeding of individuals due to the structure of the population (non-random distribution of individuals among sub-populations); *F*_ST_ also quantifies the differentiation between sub-populations in the total population (hence S and T). *F*_IT_ is a measure of the inbreeding of individuals resulting both from non-random union of gametes within sub-populations and from population structure (deviation from panmixia of all individuals of the total population, hence I and T). These *F*-statistics are classically estimated by Weir and Cockerham's unbiased estimators *f* (for *F*_IS_), *θ* (for *F*_ST_) and *F* (for *F*_IT_) [29]. When appropriate, these statistics were estimated with Fstat 2.9.3.2.

Bilateral sex-biased dispersal tests were done in Fstat 2.9.3.2 with the *F*_ST_ based test and the mean assignment index (the multilocus probability of belonging to the sampling site) corrected for population effects and its variance (*AI_c_* and *vAI_c_*) [30], as recommended in [24]. We used 10000 permutations of individuals within samples [24] and applied the tests in continental samples considering each trap containing more than three flies or all traps from the same village as the sub-population unit (two analyses).

More than three levels (i.e. individuals, sub-populations and total) exist in the tsetse samples near Dubreka. Here, individuals were sampled using traps, in sites that are located in different “districts” (i.e. Magnokhoun, Touguissoury). HierFstat version 0.03–2 [31] is an analytical package written in R [32]. This package computes hierarchical *F*-statistics from any number of hierarchical levels. The significance of *F*_T/S_, the homozygosity due to subdivision into different traps within sites, was tested by randomising individuals among traps of the same site. The significance of *F*_S/D_, which measures the relative homozygosity due to the geographical separation between sites within districts, was tested by randomizing traps (with all individuals it contains) among the different sites in the same district. Finally, *F*_D/T_ measures the relative homozygosity due to the subdivision into different districts, and was tested by randomising districts in the total sampling area. A gentle step by step description of how using HierFstat can be found in [2].

The significance of the *F*-statistics was tested by randomization (10000 permutations in each case). The significance of *F*_IS_ was tested randomising alleles between individuals within sub-samples. The significance of *F*_ST_ was tested by randomising individuals among sub-samples. These tests were performed with Fstat 2.9.3.2. For *F*_IS_ the statistic used was directly the *f* (*F*_IS_ estimator). For *F*_ST_ (and other differentiation tests), the statistic used was the maximum likelihood ratio *G* [33].

Differentiation between the northern and southern samples in Kassa were analysed by paired *F*_ST_ (*G* based test) and tested in each year (2006, 2007) with Fstat 2.9.3.2. The two *F*_ST_ were combined with an unweighted mean and the corresponding *P*-values with Fisher's procedure [34] as described in [2].

Non random association of alleles within individuals (*F*_IS_>0) may be due to null alleles. We used Micro-Checker 2.2.3 [35] to detect null alleles and estimate their frequency *p_n_* at each locus according to Brookfield's second method [36]. We compared, at each locus, the expected frequencies of blanks (i.e. null allele homozygotes) under panmixia (*p_n_* ^2^) with the blank individuals observed using a binomial exact test. For X-linked loci we compared the number of blanks observed with the expected one as computed with null allele frequency found from female data with Micro-Checker. In that case a binomial test was also undertaken with the direct null allele estimates provided by the frequency of blanks in males at such loci. For the sake of power, all binomial tests were undertaken over all sub-samples (with mean expected frequencies and total observed blanks) and were one-tailed (*H*_1_: there are fewer blanks than expected).

Short allele dominance may also explain a significant part of heterozygote deficits. It was tested using a multiple regression approach of *F*_IS_ observed at each allele at the locus of interest as a function of allele size and sub-sample, following the procedure of [37]. This was made under S-Plus 2000 professional release 2 (MathSoft Inc.). For X linked loci, only females were considered.

Because highly polymorphic microsatellite loci are used, the level of differentiation as measured by *F*_ST_ may be constrained (e.g. [2]). We thus used a “corrected” version of this statistics *F*_ST_′ = *F*_ST_/(1−*H_s_*) where *H_s_* is Nei's unbiaised estimator of genetic diversity and 1−*H_s_* corresponds to the maximum possible value for *F*_ST_ in a model with many completely isolated sub-populations (see [38]–[39], as in [40]).

The effective population size, usually noted *Ne*, is a measure of the rate at which a population looses genetic diversity by drift and roughly represent the number of adults that effectively contribute to the next generation (see [2] for a more precise definition and examples). Effective sub-population sizes could be estimated in each site using various methods: the linkage disequilibrium methods of Bartley et al. (LD_B_) [41] and of Waples and Do (LD_WD_) [42], the temporal moment based method of Waples (1989) (Temporal) [43] for sites sampled at different times, joint estimation of migration and effective population size with the maximum likelihood (ML), the moment based (Moment) methods of Wang and Whitlock [44] and the method of Vitalis and Couvet (2001) (Estim) [6], [45]–[46]. Bartley's and Waples' methods were implemented with NeEstimator [47], Waples and Do's method with LDNe [42]. Wang and Whitlock's methods were implemented with MLNE v 1.1., and Vitalis and Couvet's method was implemented by Estim 1.2 [45]. For temporal based methods six generations were assumed to separate tsetse flies in one year interval. We also estimated *m* (migration rate) from *N_e_m* with the formula *N_e_m* = (1−*F*_ST_)/(8*F*_ST_) that is appropriate for two populations and probably more appropriate here between the two Loos islands in 2006, between North and South in Kassa (2006 and 2007) and between Fotoba island and the mainland samples in 2005. We used *N_e_* from the Temporal method to extract *m*. Implementing all these methods that work under more or less different assumptions allowed the comparison of the values obtained and gaining some confidence on the parameters' range. MLNE dataset was obtained using CREATE 1.0 [48]. For LD_WD_ method, values obtained for alleles at least as frequent as 0.05 were chosen.

Signatures of bottleneck events were investigated by comparing the expected heterozygosity for a sample (*H_E_*) with the heterozygosity that would be expected for a sample taken in a population at mutation/drift equilibrium with the same size and allele number (*H_EQ_*): as allele number decreases faster than heterozygosity, bottlenecks are indicated by *H_E_*>*H_EQ_* in subsequent generations [49]. This analysis was performed using Bottleneck software [50] under an IAM (infinite allele model), a SMM (stepwise mutation model) or a TPM (two phase model), in the latter case we assumed that 70% of mutations consist of one step and 30% consist of multistep change with a variance of 30 (default values). Significance was assigned using one-tailed Wilcoxon tests [49]. Global *P*-values, overall Fotoba 2005–2006 and overall Kassa-North 2006–2007 samples were obtained with the Fisher procedure [34]. Given it only had three individuals, the Kassa-South 2006 sub-sample was excluded from these analyses. Given our sample sizes and number of loci, bottleneck detection is only possible if it occurred between *t*_1_ = 0.025×2*N_e_* and *t*_2_ = 2.5*2*N_e_* generations ago [49], where *N*_e_ represents the post-bottleneck effective population size. We compared these generation times to those believed to have occurred on Kassa Island since the 1960's (i.e. 276 generations ago) when an important bauxite mining activity is thought to have strongly altered ecological conditions (see http://www.nationsencyclopedia.com/Africa/Guinea-MINING.html). This provided a possible *N_e_* included between 55 and 5520 individuals.

### Mitochondrial markers

A portion of the 5′ end of the mitochondrial gene cytochrome oxidase 1 was amplified using the primers CI-J-2195 TTGATTTTTTGGTCATCCAGAAGT [51] and CULR TGAAGCTTAAATTCATTGCACTAATC. Double distilled water containing 10× PCR buffer (Bioline), dNTP 0.8 mM, primers 0.5 µM each, MgCl_2_ 3 mM was incubated with 0.25units of BIOTaq DNA polymerase and approximately 0.5 ng of template DNA in 25 µl reactions. Temperature cycles were 5 min 95°C, 35 cycles of 93°C for 1 min, 55°C for 1 min and 72°C for 2 min, then 72°C for 7 min. PCR products were purified using the Bioline SURECLEAN reagent (BIO-37046) according to the manufacturer's instructions, and sequenced using an ABI3730XL sequencing machine (Macrogen). Each template was sequenced bi-directionally. Sequence traces were checked using Codoncode Aligner (CodonCode Corporation), and aligned using the ClustalW algorithm implemented in MEGA version 4 with the following settings: gap opening penalty15, gap extension penalty 6.6, IUB weight matrix, transition weight 0.5, delay divergent cut-off 30 [52]. The PCR product size is 850 bp, but for analysis the alignment was trimmed to 723 bp of good quality sequence. The following statistics were calculated using DNAsp: FST_Seq was calculated according to equation 3 in [53] and is comparable to Weir and Cockerham's *F_ST_* estimator for sequence data [29]. *H*_ST_, an equivalent of Nei's estimator of *F*_ST_ (*G*_ST_) was calculated according to equation 2–4, and *K*_ST_* according to equations 7–11 in [54] and is an equivalent of Nei's sequence statistic γ_ST_ [55]. A permutation test, in which haplotypes or sequences were randomly assigned to the different localities 10000 times, was used to test the significance of *H_ST_* and *K_ST_* * [54]. Mitochondrial analysis could only be undertaken with 10 individuals from Fotoba 2005, five individuals from Touguissoury and five individuals from Magnokhoun.

## Results

The only tsetse species caught was *G. p. gambiensis*. Entomological surveys gave mean catches of flies per trap per day (FTD) of 10 in Kassa and 1 in Fotoba for Loos islands. On the mainland, mean FTDs were 7.5 in Magnokhoun, 5.5 in Touguissoury, and 11 in Falessadé.

No sex biased dispersal was found (all *P*-values>0.05) in 2005 samples from continental sites. Therefore in all further analyses data from females and males is combined.

Among the 36 tests of linkage disequilibrium between paired loci (locus XB110 was excluded due to insufficient polymorphism), only two pairs were in significant linkage that did not stay significant after Bonferroni correction (*P*_Binomial, 2, 36, 0.05_ = 0.5433).

HierFstat analyses gave no effect for district (*F*_D/T_ = 0.005, *P* = 0.21), site (*F*_S/D_ = 0.016, *P* = 0.454) or traps (*F*_T/S_ = −0.006, *P* = 0.90). Thus, individual tsetse flies from Touguissoury and Magnokhoun (Dubréka focus) were considered to belong to the same population for the following analyses. In Kassa, over 2006 and 2007 a substantial differentiation could be seen between northern and southern samples (*F*_ST_ = 0.095, *P* = 0.018).

**Figure 2:**
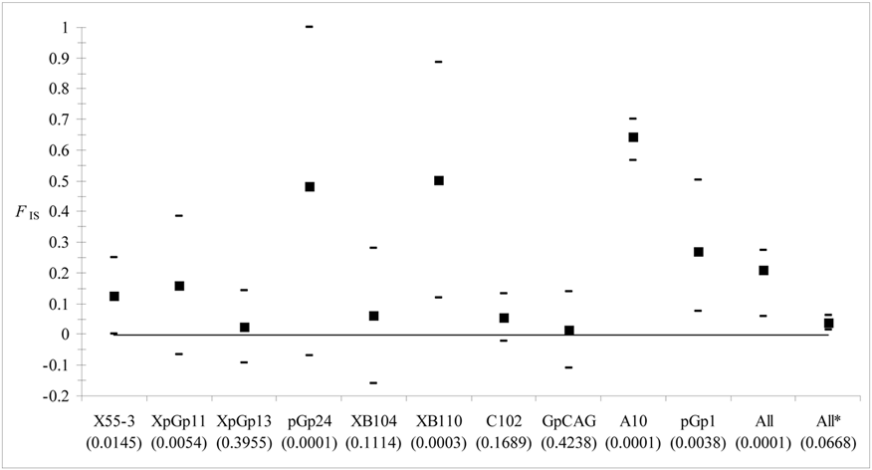
*F*_IS_ values for each locus and over all loci. Confidence intervals were obtained after jackknifing over samples, except for overall loci obtained by bootstrap over loci, for loci pGp1 and A10 that were only available in Kassa 2007 samples (two values that provide the range) and for the mean *F*_IS_ over the four loci where no null alleles were evidenced and for which the range is given by the minimum and maximum values observed. *P*-values obtained after testing that *F*_IS_ is not significantly above 0 (10000 permutations) are presented between brackets.

*F*_IS_ analysis revealed a significant excess of homozygosity, variable across loci and significant for X55.3, XpGp11, pGp24, XB110, pGp1 and A10 (Figure 2). All but XB110 were reasonably explained by the presence of null alleles (Table 1). For XB110, the binomial test was only significant with “Males” method, which could be explained by Type I error, given the number of tests undertaken. There was no evidence for short allele dominance at this locus. Excluding the six loci with null alleles provided a much smaller deviation of heterozygote frequency from panmictic expectation (*F*_IS_ = 0.04, *P* = 0.069) (Figure 2).

**Table 1:** Results obtained during Microchecker analyses.

Loci with positive *F*_IS_	Observed blanks	Total sample size	Expected blank frequency			
			Brookfield 2	*P*-value	Males	*P*-value
pGp24	54	192	0.315	0.179	ND	ND
A10	3	30	0.149	0.329	ND	ND
pGp1	1	30	0.032	0.754	ND	ND
X55.3	9	109	0.045	0.975	0.039	0.989
XpGp11	19	109	0.137	0.893	0.026	1
XB110	3	109	0.033	0.516	0.076	**0.030**

The six loci that gave positive results are presented with the total number of genotyped individuals for each locus (blanks included). The sum of expected blanks over all samples divided by the sample size gave the expected frequency of blanks and the result of the exact binomial test comparing the observed proportion of blanks to the expected one (H1: there are less blanks than expected with null alleles). Expected frequencies were computed with Brookfield's second method for all loci or directly with the proportion of blank males at X-linked loci as described in the Methods section. Significant test (in bold) means that not enough blank individuals were observed at that locus for null alleles to explain the observed *F*_IS_.

Differentiation was significantly positive between each continental site and Loos islands (2005 samples, *P*<0.0001). In Table 2 are presented the paired *F*_ST_, mean 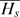 between paired samples and paired 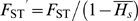. The highest levels of differentiation were found between Loos islands and all the other sites. Geographical differentiation was highly significant for each pair of samples and the smallest (although highly significant) value was observed between continental sub-samples (Falessadé and Dubréka). Within the Loos islands, there was a high and significant differentiation between Kassa and Fotoba, the two main islands. It is noteworthy that the same analysis undertaken with the four loci without null alleles (XpGp13, XB104, C102 and GpCAG) provided very similar results (not shown), with the exception of the temporal analysis for which the weak differentiation observed in Kassa between 2006 and 2007 was no longer significant. Genotypes at microatellite loci of all the individuals analysed can be seen in Table S1.

**Table 2:** Differentiation between paired samples of tsetse flies from Guinea as measured by *F*_ST_ (Weir and Cockerham estimator) and 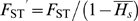, corrected for polymorphism with Nei's genetic diversity averaged over the two compared samples.

	Sub-samples	*F*_ST_	*P*-value	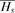	*F*_ST_′
**Geographical**	Falessadé and Dubréka 2005	0.0188	0.0001	0.6991	0.0625
	Falessadé and Fotoba 2005	0.0715	0.0001	0.6086	0.1827
	Dubréka and Fotoba 2005	0.0971	0.0001	0.6055	0.2461
	Fotoba and Kassa North 2006	0.0989	0.0014	0.4963	0.1963
	Fotoba and Kassa South 2006	0.1380	0.0158	0.4200	0.2379
	Kassa North and South (2006, 2007)	0.0854	0.0119	0.5620	0.1949
**Temporal**	Fotoba 2005 and 2006	0.0240	0.0441	0.4900	0.0471
	Kassa North 2006 and 2007	0.0242	0.1655	0.5612	0.2272
	Kassa South 2006 and 2007	0.1683	0.0271	0.4548	0.3087

Both geographical (contemporaneous sub-samples) and temporal (sympatric sub-samples) are provided and *P*-values are also given (all significant except between Kassa North 2006 and 2007). For Kassa North and South (2006, 2007) *F*_ST_ was averaged over years and the corresponding *P*-value obtained with Fisher's procedure.

A fragment of the mitochondrial Cytochrome Oydase I gene was amplified by PCR and sequenced (see Table S2). COI sequences were deposited in Genbank (accession numbers FJ387505-FJ387524). The statistics of genetic differentiation based on COI are presented in Table 3. Within Dubréka, as with nuclear microsatellite markers, Touguissoury 2005 and Magnokhoun 2005 showed an absence of differentiation. Differentiation between Fotoba and Dubréka was strong and significant (Table 3) and comparable to that observed using microsatellites (Table 2).

**Table 3:** Statistics on comparisons of mitochondrial cytochrome oxidase 1 sequences (723 sites in alignment, no gaps) for 2005 samples of *G. palpalis gambiensis*.

Population pair	*F*_ST_Seq_	*H*_ST_	*K*_ST_*
Fotoba (n = 10)	0.148	**0.144 (0.009)**	**0.109 (0.029)**
Touguissoury (n = 5)			
Fotoba (n = 10)	0.043	**0.114 (0.027)**	0.087 (0.072)
Magnokhoun (n = 5)			
Touguissoury (n = 5)	−0.113	0.000 (1)	−0.035 (0.829)
Magnokhoun (n = 5)			
Fotoba (n = 10)	0.124	**0.101 (0.008)**	**0.084 (0.016)**
Dubréka (n = 10)			

*P*-values are given between brackets. Significant tests are in bold.

Effective population size estimates with different methods are given in Table 4. These results were obtained for all loci, including those with null alleles. Using the four loci without null alleles or even the two autosomal loci with no deviation from Hardy-Weinberg (C102 and GpCAG) greatly widened confidence intervals but provided mean estimates of the same order of magnitude as with the complete data set (not shown). Table 5 gives migration rate (*m*) estimates for the three sites (Fotoba, Kassa North and Kassa South) for which it was possible. Except for LD_WD_ and Estim for which most *N_e_* were infinite or undefined, the different methods gave consistent *N_e_* and *m* estimates, particularly for Fotoba where the four available methods converged to very similar estimates. On the mainland, Dubréka seemed to harbour a large effective population of tsetse flies (475<*N_e_*<2016) while Falessadé showed a notably small estimate (25<*N_e_*<63). Loos islands displayed surprisingly small effective population sizes (Table 4, 2<*N_e_*<145).

**Table 4:** Effective population size (*N_e_*) of the different samples with 95% confidence intervals (CI) obtained with five different methods (when possible).

	LD_B_	LD_WD_	Temporal	ML	Estim
Dubréka 2005	2016 (313, ∞)	475 (75, ∞)			∞ (∞, ∞)
Falessadé 2005	25 (19, 35)	63 (21, ∞)			∞ (0, ∞)
Fotoba 2005	145 (38, ∞)	∞ (26, ∞)			24 (2, ∞)
Fotoba 2006	35 (6, 383)	53 (9, ∞)			∞ (2, ∞)
Fotoba	90 (22, ∞)	∞ (18, ∞)	42 (17, 134)	37 (18, 86)	∞ (2, ∞)
Kassa North 2006	11 (7, 18)	∞ (32, ∞)			0 (0, ∞)
Kassa North 2007	22 (14, 46)	∞ (∞, ∞)			0 (0, ∞)
Kassa North	17 (11, 32)	∞ (∞, ∞)	35 (14, 107)	34 (16, 105)	0 (0, ∞)
Kassa South 2006	2 (1, 6)	∞ (∞, ∞)			∞ (0, ∞)
Kassa South 2007	2 (2, 2)	∞ (∞, ∞)			∞ (0, ∞)
Kassa South	2 (1, 4)	∞ (∞, ∞)	11 (4, 46)	11 (5, 35)	∞ (0, ∞)

For Fotoba and Kassa, sequential values obtained with LD methods were also averaged. Moment based method from Wang and Whitlock did not provide any *N_e_*.

∞: infinity.

**Table 5:** Estimation of migration rates with three methods on the three sites where this was possible.

	Methods			
	ML	Moment	Estim	*N_e_m*/*N_e_*Temporal
Fotoba (*N_e_m* = 1.18)	0.024 [0.009, 0.056]	0.020	0.031	0.032
Kassa North (*N_e_m* = 1.24)	0.043 [0.014, 0.104]	0.070		0.036
Kassa South (*N_e_m* = 1.06)	0.172 [0.052, 0.551]	0.430		0.096

*N_e_m* estimates, based on two population model, were averaged over estimates from Table 2 and *m* extracted using the Temporal estimate of *N_e_* from Table 4. Neither Moment or Estim methods provided confidence intervals.

Bottleneck signatures were only detected with the IAM model in Kassa North sub-sample with a global *P*-value (Fisher's procedure) of 0.022. Thus, bottleneck detection in Kassa North is suggestive of a post bottleneck effective population size range (55-5520) that overlaps with what was suggested by other methods, most particularly with temporal based methods (Table 4), but with of course a much higher upper bound.

## Discussion

Both microsatellite and mitochondrial DNA markers revealed a strong barrier to gene flow between *G. p. gambiensis* samples from the Loos islands and continental areas. Even within the Loos islands, the genetic differentiation was significant. For the first time in tsetse flies within the palpalis group, we provide estimates of effective population sizes. In the Loos islands these estimates were found to be low despite high observed densities of tsetse.

### Genetic differentiation between pairs of populations: implications for control

The high and significant genetic differentiation found between the Loos islands and the mainland combined with the small total surface area to be treated (around 15 sq km), has led the Guinean National Control Programme to launch a tsetse elimination campaign (M. CAMARA, pers. com.). We show here that within the Loos islands, the high and significant differentiation found between Fotoba and Kassa suggests a low number of migrants between islands (probably less than one per generation). This result, combined with morphometric data suggest that the elimination can be based on the implementation of an area wide approach using a sequential control strategy on each of the islands in turn [16],[40]. On the mainland, all sites in Dubréka could be considered as within a single reproductive unit. These sites were significantly differentiated but not completely isolated, from Falessadé (Savannah site). This suggests that, contrarily to what was observed on Loos islands, tsetse elimination in this mainland area should not yet be chosen as the ideal strategy so far because exchanges of flies between these sites may still occur through the dense hydrographic network present. An elimination programme in the area would then require to create artificial barriers around the area to be treated (made of insecticide impregnated traps for instance, see [56]), which would prevent immigration into this area. Such a programme would require more detailed sampling. Alternatively, tsetse control with the participation of local communities to reduce (but not eliminate) tsetse densities may be advised since this has been shown to be technically feasible [57].

### *G. palpalis* structure within populations

Over all sample sites and loci in our Guinean samples, the strong and significant *F*_IS_ found was reasonably explained by null alleles, it was thus not necessary to invoke a Wahlund effect, in contrast to results obtained in Burkina Faso for the same tsetse species [22],[58] or for the closely related subspecies *G. p. palpalis* in Côte d'Ivoire [59]. In Guinea, this could be explained by the high rainfall (3000 mm /annum) and large numbers of river and stream habitats, combined with good host availability allowing good dispersal conditions and a less restricted distribution. The exception to this would be on Kassa, where a human settlement that led to habitat fragmentation may have caused a slight Wahlund effect (in this island mean *F*_IS_ = 0.092, *P* = 0.058), which may also explain why *N_e_* estimated with LD methods were often lower than those estimated with temporal based methods.

We found surprisingly low estimates of *N_e_*. To our knowledge, such estimates were made only once in tsetse, for a savannah species belonging to the *morsitans* group in East Africa, *G. swynnertoni* [60], which is phylogenetically and ecologically very far from *G. palpalis* (riverine fly of the palpalis group) [61]. Using mitochondrial markers, Marquez *et al.*, 2006 found very low estimates of population size, and attributed it to a recent bottleneck. In Kassa Island, tsetse seemed structured in fairly isolated populations that may explain why extremely small *N_e_* were found. However, Kassa Island is the place where tsetse densities were the highest (mean of 10 flies / trap / day, up to 100 in some traps). In North Kassa, the possible signature for a Bottleneck that occurred about 276 generations ago led to a higher estimate of post bottleneck effective population size (between 55 and 5520). The only explanation we can provide is that intense bauxite mining activity in Kassa caused a drastic reduction in *G. p. gambiensis* populations at that time (year 1960 corresponds to 276 generations from 2006 sampling using 6 generations per year). Indeed it is very well known that bauxite mining is very destructive for the environment (e.g. http://www.idrc.ca/en/ev-31010-201-1-DO_TOPIC.html). In contrast on Fotoba, which was not involved in bauxite mining activity, no bottleneck could be detected. After the end of these mining activities in Kassa, tsetse populations would have recovered, and reached high densities again, aided by the important pig rearing activity which began on Kassa (but not in Fotoba) since the 1980s. A significant correlation has been observed in Kassa between tsetse densities and pig rearing presence [62].

No signature of a bottleneck was found in any other site. Reduction in effective population size can occur in the case of variance in reproductive success. In the case of tsetse flies, where a modal number of four larval progeny per female can be assumed, there is more opportunity for variance in male mating success. In Appendix S1 we have derived a very simple model aimed at illustrating the drop in *N_e_* as a function of the census size *N_c_* and number of sired females per successful males *nf_fec_* that would result when some males sire several females while the others do not. We used North Kassa estimate of “real” *N_e_*, obtained from the bottleneck procedure, to estimate a possible range for *nf_fec_* using equation (5) from Appendix S1. This estimate ranged between four and 490 females mated per successful males. Table 6 provides the results obtained for *N_c_* in each sub-sample when applying equation (6) of Appendix S1. Apparently, variance in male reproductive success must be very high if this has to explain all small *N_e_*. A variance in female reproductive success may also act but is less probable since when females were dissected, most of them were found to be pregnant (tsetse are viviparous) (data not shown). Whatever the cause of these small effective population sizes, they are suggestive of significant (very high in certain sites such as in Kassa Island) levels of inbreeding in *G. palpalis gambiensis* populations from coastal Guinea.

**Table 6:** Estimation of the possible range for population sizes (*N_c_*) as a function of the number of females sired by the most successful males (other males do not mate) (*nf_fec_*) in order to explain small effective population sizes found in Guinean tsetse flies.

Sub-sample	Order of magnitude of *N_e_*	*N_c_* if *nf_fec_* = 4	*nf_fec_* = 460
Dubréka	1000	2467	245333
Falessadé	40	99	9813
Fotoba	60	148	14720
Kassa North	30	74	7360
Kassa South	10	25	2453

Details for computations can be found in the text and Appendix S1. The Order of magnitude of *N_e_* for each subsample comes from Table 4.

These results suggest that further analyses should be conducted on the potential of *G. p. gambiensis* to maintain genetic diversity at local and global scales in Guinea, in particular regarding interactions with the aetiological agent of HAT, *Trypanosoma brucei gambiense*, the epidemiology of which is known to vary substantially through West Africa and in Guinea in particular [13],[15]. We also hope that, within the context of the Pan African eliminations programmes that have been launched by the WHO [11]–[12] and the African Union PATTEC (Pan African Tsetse and Trypanosomosis Eradication Campaign, [63]), more studies will be conducted on tsetse population genetics. Indeed, control programmes are beginning to recognise the importance of such data in helping to choose specific control strategies.

## Supporting Information

Alternative Language Abstract S1 Translation of the Abstract into French by Philippe Solano.
(0.03 MB DOC)

Appendix S1 (0.03 MB DOC)

Table S1 (0.13 MB XLS)

Table S2 (0.03 MB DOC)

## References

[1] Criscione CD, Blouin MS (2005). Effective sizes of macroparasite populations: a conceptual model.. Trends Parasitol.

[2] De Meeûs T, McCoy KD, Prugnolle F, Chevillon C, Durand P (2007). Population genetics and molecular epidemiology or how to “débusquer la bête”.. Infect Genet Evol.

[3] Koffi BB, De Meeûs T, Barré N, Durand P, Amathau C (2006). Founder effects, inbreeding and effective sizes in the Southern cattle tick: the effect of transmission dynamics and implications for pest management.. Mol Ecol.

[4] Prugnolle F, De Meeûs T, Durand P, Sire C, Théron A (2002). Sex-specific genetic structure in *Schistosoma mansoni*: evolutionary and epidemiological implications.. Mol Ecol.

[5] Slatkin M (1987). Gene flow and the geographic structure of natural populations.. Science.

[6] Vitalis R, Couvet D (2001c). Two-locus identity probabilities and identity disequilibrium in a partially selfing subdivided population.. Genet Res.

[7] Watts PC, Rousset F, Saccheri IJ (2007). Compatible genetic and ecological estimates of dispersal rates in insect (*Coenagrion mercuriale*: Odonata: Zygoptera) populations: analysis of ‘neighbourhood size’ using a more precise estimator.. Mol Ecol.

[8] Milgroom MG (1996). Recombination and the multilocus structure of fungal populations.. Annu Rev Phytopathol.

[9] Taylor JW, Geiser DM, Burt A, Koufopanou V (1999). The evolutionary biology and population genetics underlying fungal strain typing.. Clin Microbiol Rev.

[10] Tibayrenc M (1999). Towards an integrated genetic epidemiology of parasitic protozoa and other pathogens.. Annu Rev Genet.

[11] Jannin JG (2005). Sleeping sickness-a growing problem?. Br Med J (Clin Res Ed).

[12] Simarro PP, Jannin J, Cattand P (2008). Eliminating Human African Trypanosomiasis: where do we stand and what comes next?. PLoS Med.

[13] Courtin F, Jamonneau V, Duvallet G, Garcia A, Coulibaly C (2008). Sleeping sickness in West Africa (1906–2006): Changes in spatial repartition and lessons from the past.. Trop Med Int Hlth.

[14] Brengues J, Challier A, Ouedraogo VK (1964). *Contribution à la connaissance de l'épidémiologie de la trypanosomiase humaine: enquête entomologique dans les territoires kissis et limitrophes (avril-mai 1964: Sierra-Leone, Libéria, Guinée)* OCCGE, Bobo-Dioulasso, Burkina Faso.

[15] Camara M, Kaba D, KagbaDouno M, Sanon JR, Ouendeno F (2005). Human African trypanosomiasis in the mangrove forest in Guinea: epidemiological and clinical features in two adjacent outbreak areas.. Med Trop.

[16] Vreysen M, Robinson AS, Hendrichs J (2007). Area wide control of insect pests.

[17] Laveissiere C, Grebaut P (1990). Recherches sur les pièges a glossines (*Diptera*: *Glossinidae*). Mise au point d'un modèle économique: le piège “Vavoua”.. Trop Med Parasitol.

[18] Solano P, Duvallet G, Dumas V, Cuisance D, Cuny G (1997). Microsatellite markers for genetic population studies in *Glossina palpalis* (Diptera: Glossinidae).. Acta Trop.

[19] Luna C, Bonizzoni M, Cheng Q, Robinson AS, Aksoy S, Zheng L (2001). Microsatellite polymorphism in tsetse flies.. J Med Entomol.

[20] Baker MD, Krafsur ES (2001). Identification and properties of microsatellite markers in tsetse flies *Glossina morsitans* sensu lato (Diptera: Glossinidae).. Mol Ecol Notes.

[21] Gooding RH, Solano P, Ravel S (2004). X-chromosome mapping experiments suggest occurrence of cryptic species in the tsetse fly *Glossina palpalis palpalis*.. Can J Zool.

[22] Solano P, De La Rocque S, De Meeûs T, Cuny G, Duvallet G (2000). Microsatellite DNA markers reveal genetic differentiation among populations of *Glossina palpalis gambiensis* collected in the agro-pastoral zone of Sideradougou, Burkina Faso.. Insect Mol Biol.

[23] Walsh PS, Metzger DA, Higuchi R (1991). Chelex 100 as a medium for simple extraction of DNA for PCR-based typing from forensic material.. Biotechniques.

[24] Goudet J, Perrin N, Waser P (2002). Tests for sex-biased dispersal using bi-parentally inherited genetic markers.. Mol Ecol.

[25] Goudet J (1995). FSTAT (Version 1.2): A computer program to calculate F-statistics.. J Hered.

[26] Holm S (1979). A simple sequentially rejective multiple test procedure.. Scand J Stat.

[27] Prugnolle F, De Meeûs T (2002). Inferring sex-biased dispersal from population genetic tools: a review.. Heredity.

[28] Hartl DL, Clark AG (1989). Principles of Population Genetics.

[29] Weir BS, Cockerham CC (1984). Estimating F-statistics for the analysis of population structure.. Evolution.

[30] Favre L, Balloux F, Goudet J, Perrin N (1997). Female-biased dispersal in the monogamous mammal *Crocidura russula*: evidence from field data and microsatellite patterns.. Proc R Soc London B.

[31] Goudet J (2005). HIERFSTAT, a package for R to compute and test hierarchical F-statistics.. Mol Ecol Notes.

[32] R-Development-core-team (2008). R: A Language and Environment for Statistical Computing.. http://www.R-project.org.

[33] Goudet J, Raymond M, De Meeûs T, Rousset F (1996). Testing differentiation in diploid populations.. Genetics.

[34] Fisher RA (1970). Statistical Methods for Research Workers.

[35] Van Oosterhout C, Hutchinson WF, Wills DPM, Shipley P (2004). MICRO-CHECKER: software for identifying and correcting genotyping errors in microsatellite data.. Mol Ecol Notes.

[36] Brookfield JFY (1996). A simple new method for estimating null allele frequency from heterozygote deficiency.. Mol Ecol.

[37] De Meeûs T, Humair PF, Grunau C, Delaye C, Renaud F (2004). Non-Mendelian transmission of alleles at microsatellite loci: an example in *Ixodes ricinus*, the vector of Lyme disease.. International Journal for Parasitology.

[38] Hedrick PW (1999). Perspective: Highly variable loci and their interpretation in evolution and conservation.. Evolution.

[39] Hedrick PW (2005). A standardized genetic differentiation measure.. Evolution.

[40] Camara M, Caro-Riano H, Ravel S, Dujardin JP, Hervouet JP (2006). Genetic and morphometric evidence for population isolation of *Glossina palpalis gambiensis* (Diptera: Glossinidae) on the Loos islands, Guinea.. J Med Entomol.

[41] Bartley D, Bagley M, Gall G, Bentley B (1992). Use of linkage disequilibrium data to estimate effective size of hatchery and natural fish populations.. Conserv Biol.

[42] Waples RS, Do C (2008). LDNE: a program for estimating effective population size from data on linkage disequilibrium.. Molecular Ecology Resources.

[43] Waples RS (1989). A generalized approach for estimating effective population size from temporal changes in allele frequency.. Genetics.

[44] Wang JL, Whitlock MC (2003). Estimating effective population size and migration rates from genetic samples over space and time.. Genetics.

[45] Vitalis R, Couvet D (2001). ESTIM 1.0: a computer program to infer population parameters from one- and two-locus gene identity probabilities.. Mol Ecol Notes.

[46] Vitalis R, Couvet D (2001). Estimation of effective population size and migration rate from one- and two-locus identity measures.. Genetics.

[47] Peel D, Ovenden JR, Peel SL (2004). NeEstimator: software for estimating effective population size, Version 1.3.. Queensland Government, Department of Primary Industries and Fisheries.

[48] Coombs JA, Letcher BH, Nislow KH (2008). CREATE: a software to create input files from diploid genotypic data for 52 genetic software programs.. Mol Ecol Res.

[49] Cornuet JM, Luikart G (1996). Description and power analysis of two tests for detecting recent population bottlenecks from allele frequency data.. Genetics.

[50] Piry S, Luikart G, Cornuet JM (1999). BOTTLENECK: A computer program for detecting recent reductions in the effective population size using allele frequency data.. J Hered.

[51] Simon C, Frati F, Bechenbach A (1994). Evolution, weighting, and phylogenetic utility of mitochondrial gene sequences and a compilation of conserved polymerase chain reaction primers.. Ann Entomol Soc Am.

[52] Tamura K, Dudley J, Nei M, Kumar S (2007). MEGA4: Molecular Evolutionary Genetics Analysis (MEGA) software version 4.0.. Mol Biol Evol.

[53] Hudson RR, Slatkin M, Maddison WP (1992). Estimation of levels of gene flow from DNA sequence data.. Genetics.

[54] Hudson RR, Boos DD, Kaplan NL (1992). A statistical test for detecting geographic subdivision.. Mol Biol Evol.

[55] Nei M (1987). Molecular Evolutionary Genetics.

[56] Cuisance D, Politzar H (1983). Etude sur l'efficacité contre *Glossina palpalis gambiensis* et *G. tachinoides* de barrières constituées d'écrans ou de pièges biconiques imprégnés de DDT, de deltaméthrine, ou de dieldrine.. Rev Elev Méd Vét Pays trop.

[57] Laveissière C, Penchenier L (2005). Manuel de lutte contre la maladie du sommeil.

[58] Bouyer J, Ravel S, Dujardin JP (2007). Population structuring of *Glossina palpalis gambiensis* (Diptera: Glossinidae) according to landscape fragmentation in the Mouhoun river, Burkina Faso.. J Med Entomol.

[59] Ravel S, De Meeûs T, Dujardin JP, Zézé GD, Gooding RH (2007). The tsetse fly *Glossina palpalis palpalis* is composed of several genetically differentiated small populations in the sleeping sickness focus of Bonon, Côte d'Ivoire.. Infect Genet Evol.

[60] Marquez JG, Malele I, Ouma JO, Krafsur ES (2006). *Glossina swynnertoni* (Diptera: Glossinidae): effective population size and breeding structure estimated by mitochondrial diversity.. Bull Entomol Res.

[61] Dyer NA, Lawton SP, Ravel S, Choi KS, Lehane MJ, Robinson AS, Okedi LM, Hall M, Solano P, Donnelly MJ (2008). Molecular phylogenetics of tsetse flies (*Diptera*: *Glossinidae*) based on mitochondrial (CO1, 16S, ND2) and nuclear ribosomal DNA sequences, with an emphasis on the *palpalis* group.. Mol Phyl Evol.

[62] Kagbadouno M, Camara M, Bouyer J, Hervouet JP, Morifaso O, Kaba D, Jamonneau V, Solano P (2009). Tsetse elimination: its interest and feasibility in the historical sleeping sickness focus of Loos islands, Guinea.. Parasite.

[63] Schofield CJ, Kabayo J (2008). Trypanosomiasis vector control in Africa and Latin America.. Paras Vect.

