# Supplementary material for: The Population Structure of Glossina palpalis gambiensis from Island and Continental Locations in Coastal Guinea
Source: PLoS Negl Trop Dis. 2009 Mar 17;3(3):e392. doi: 10.1371/journal.pntd.0000392 (PMC2652410; doi:10.1371/journal.pntd.0000392)
Supplement: Alternative Language Abstract S1 — Translation of the Abstract into French by Philippe Solano. (0.03 MB DOC) [file pntd.0000392.s001.doc]

**Résumé** (translation of the abstract by Philippe Solano)

Nous avons mené une étude de génétique des populations grâce à des marqueurs microsatellites et mitochondriaux sur le vecteur de maladie du sommeil le plus dangereux d’Afrique de l’Ouest, *Glossina palpalis gambiensis*. L’objectif était de connaître la taille efficace des populations et de mesurer le degré d’isolement génétique :

- Entre les populations de la mangrove continentale (foyer de Dubréka) et celles des îles de Loos,
- Entre les populations de chacune des îles de Loos, et
- au sein du foyer de Dubréka entre les populations de savane et celles de mangrove,

afin de pouvoir en déduire les stratégies de lutte anti-vectorielle les mieux adaptées selon le biotope et les résultats.

Nous avons mis en évidence une forte et significative différenciation génétique entre les tsé-tsé des îles de Loos et celles du foyer de Dubréka. Cette différenciation existe également entre les différentes îles de Loos (Kassa et Fotoba), avec peu de migration entre ces îles. Sur le continent, la population du foyer de Dubréka apparaît panmictique, mais les échanges avec les populations de savane sont peu fréquents. De manière inattendue, les effectifs efficaces apparaissent petits, surtout sur les îles de Loos. Sur l’île de Kassa, un « goulot d’étranglement » semble être survenu en raison des activités de bauxite menées dans le passé sur cette île. Pour les autres sites, d’autres explications doivent être recherchées, notamment une possible variance dans le succès de la reproduction.

Nos résultats suggèrent qu’une stratégie d’élimination, menée séquentiellement, peut être envisagée sur les îles de Loos. Dans le foyer de Dubréka, les tailles importantes de population et les échanges fréquents suggèrent que, sur la base de ces informations actuelles, le contrôle (et non pas l’élimination totale) en vue de réduire la transmission de maladie du sommeil peut être conseillé.
