# Supplementary material for: The Population Structure of Glossina palpalis gambiensis from Island and Continental Locations in Coastal Guinea
Source: PLoS Negl Trop Dis. 2009 Mar 17;3(3):e392. doi: 10.1371/journal.pntd.0000392 (PMC2652410; doi:10.1371/journal.pntd.0000392)
Supplement: Appendix S1 — (0.03 MB DOC) [file pntd.0000392.s002.doc]

**Appendix 1**

Let *N_f_* and *N_m_* be the number of female and male adults respectively. Then, the effective population size is known as (e.g. (Hartl, Clark, 1989) page 86):

$N_{e}=\frac{4N_{f}N_{m}}{\left(N{}_{f}+N_{m}\right)}$ (1)

The total number of adults (census size) *N_c_*=2*N_f_* if a balanced sex ratio is assumed.

Let $nf_{fec}$ be the number of females sired by each most successful males and *x* the number of most successful males (other males do not mate successfully), then:

$x=\frac{N_{f}}{nf_{fec}}=\frac{N_{c}}{2nf_{fec}}$ (2)

Combining (1) and (2) gives:

$N_{e}=\frac{4\frac{N_{c}}{2nf_{fec}}\frac{N_{c}}{2}}{\left(\frac{N_{c}}{2nf_{fec}}+\frac{N_{c}}{2}\right)}=\frac{\frac{N_{c}^{2}}{nf_{fec}}}{\frac{N_{c}(1+nf_{fec})}{2nf_{fec}}}$ (3)

For all *nf_fec_*>0 and all *N_c_*>0 equation (3) reduces to:

$N_{e}=\frac{2N_{c}}{1+nf_{fec}}$ (4)

Then if *N_c_* is known, we can extract *nf_fec_* as:

$nf_{fec}=\frac{2N_{c}}{N_{e}}-1$ (5)

or if *nf_fec_* is known, we can extract *N_c_* as:

$N_{c}=\frac{1+nf_{fec}}{2}N_{e}$ (6)
