# Supplementary material for: The Population Structure of Glossina palpalis gambiensis from Island and Continental Locations in Coastal Guinea
Source: PLoS Negl Trop Dis. 2009 Mar 17;3(3):e392. doi: 10.1371/journal.pntd.0000392 (PMC2652410; doi:10.1371/journal.pntd.0000392)
Supplement: Table S2 — (0.03 MB DOC) [file pntd.0000392.s004.doc]

### Table S2.

|  | Fotoba 2005 | Touguissory 2005 | Magnokhoun 2005 | Magnokhoun and Touguissory 2005 | Total |
| --- | --- | --- | --- | --- | --- |
| Number of individuals sequenced | 10 | 5 | 5 | 10 | 20 |
| Number of haplotypes | 3 | 5 | 5 | 10 | 12 |
| Haplotypes shared with other populations | 1 (with Magnokhoun) | 0 | 1 (with Fotoba) | 1 (with Fotoba) | 1 |
